# Breast Cancer and Fertility Preservation in Young Female Patients: A Systematic Review of the Literature

**DOI:** 10.3390/clinpract13060127

**Published:** 2023-11-13

**Authors:** Ioannis Boutas, Adamantia Kontogeorgi, Nektarios Koufopoulos, Dionysios T. Dimas, Kyparissia Sitara, Sophia N. Kalantaridou, Constantine Dimitrakakis

**Affiliations:** 1 Breast Unit, Rea Maternity Hospital, P. Faliro, 17564 Athens, Greece; 2 Third Department of Obstetrics and Gynecology, Attikon University Hospital, National and Kapodistrian University of Athens, Rimini 1, 12462 Chaidari, Greece; medp2012031@med.uoc.gr (A.K.); skalanta@med.uoa.gr (S.N.K.); 3 Second Pathology Department, Attikon University Hospital, National and Kapodistrian University of Athens, Rimini 1, 12462 Chaidari, Greece; nkoufo@med.uoa.gr; 4 Breast Unit, Athens Medical Center, Psychiko Clinic, 11525 Athens, Greece; dionysis.dimas@gmail.com; 5 Department of Internal Medicine, “Elpis” General Hospital, 11522 Athens, Greece; kdsitara@gmail.com; 6 First Department of Obstetrics and Gynecology, Alexandra University Hospital, National and Kapodistrian University of Athens, Lourou 4-2, 11528 Athens, Greece; dimitrac@ymail.com

**Keywords:** breast cancer, fertility preservation, cryopreservation, GNRH analogs

## Abstract

Introduction: Breast cancer affects almost 1.5 million women worldwide below the age of 45 years each year. Many of these women will be advised to undergo adjuvant chemotherapy to minimize the risk of death or recurrence of the tumor. For these patients, chemotherapy is a known cause of infertility, as it can damage primordial follicles, which can lead to early menopause or premature ovarian insufficiency. This systematic review aims to synthesize the current evidence of the most suitable treatments for fertility preservation. Methodology: This review was performed following the PRISMA guidelines. The authors conducted an extensive search from the last 15 years. Relevant studies were pursued in PubMed, Embase, and the Cochrane Library up until 31 July 2023. A total of seven eligible studies were identified. Results: From the reviewed literature, ovarian suppression with gonadotropin-releasing hormone agonists showed promising results in preserving fertility for breast cancer patients undergoing chemotherapy. Additionally, oocyte and embryo cryopreservation demonstrated successful outcomes, with embryo cryopreservation being the most effective option. Notably, the slow-freezing and vitrification methods were both effective in preserving embryos, with vitrification showing superior results in clinical-assisted reproductive technologies. Ovarian tissue cryopreservation emerged as a viable option for prepubertal girls and those unable to undergo conventional ovarian stimulation. The potential of in vitro maturation (IVM) as an alternative method presents a promising avenue for future fertility preservation research. Discussion: The most suitable treatments for fertility preservation in young patients is the temporary suppression with luteinizing hormone-releasing analogs, while the patient undergoes chemotherapy and cryopreservation. For cryopreservation, the physicians might deem it necessary to either cryopreserve ovarian tissue taken from the patient before any treatment or cryopreserve embryos/oocytes. Cryopreservation of oocytes and/or embryos is the most effective solution for fertility preservation in women of reproductive age, who have a sufficient ovarian reserve and are diagnosed with breast cancer, regardless of the histological type of the tumor. Because approximately 50% of young breast cancer patients are interested in becoming pregnant right after completion of therapy, the evolution and development of fertility preservation techniques promise to be very exciting.

## 1. Introduction

For female patients, breast cancer is the most common malignancy worldwide. Almost 1.5 million women below the age of 45 every year are breast cancer patients. This is about 11% of the total reported cancer cases per year [1,2,3]. These tumors tend to begin from ductal hyperproliferation and can either grow into tumors of a benign nature or aggressive metastatic carcinomas. It is worth mentioning that young patients have a higher risk of developing more aggressive carcinomas [4,5]. Breast cancer has a multitude of risk factors that can increase the possibility of developing the disease, including age, sex, family history, unhealthy lifestyle, gene mutations, or even hormone replacement therapy [6].

For a patient diagnosed with breast cancer at a young age, fertility and pregnancy issues that might arise during treatment contribute to their emotional and psychological distress [7]. Physicians should address these issues early and intervene promptly after the diagnosis to positively affect the outcome of the treatment and the long-term quality of life for these women [8]. If the disease is diagnosed early, it has a high survival rate and a good prognosis [9]; therefore, guidelines internationally recommend that physicians should discuss in the early stages of the disease informing young patients of the potential risks that they might suffer during treatment and discuss potential solutions for their fertility preservation [10,11,12]. Cancer treatment can damage primordial follicles, which can lead to early menopause or premature ovarian insufficiency.

The number of primordial follicles, also known as ovarian reserve, plays a substantial role in a patient’s fertility status [13]. To tackle the problem, physicians need to present sustainable resolutions to the problem. One of the most suitable treatments for fertility preservation in these patients is the temporary suppression with luteinizing hormone-releasing analogs (LHRHas), while the patient undergoes chemotherapy and cryopreservation. For cryopreservation, the physicians might deem it necessary to either cryopreserve ovarian tissue taken from the patient before any treatment or cryopreserve embryos/oocytes [14]. Something of great interest is that after young patients are diagnosed with breast cancer, approximately 50% of them are interested in becoming pregnant right after completion of therapy. Unfortunately, breast cancer survivors have the lowest possibility for a subsequent pregnancy. This is due to the gonadotoxic therapeutic approach and the prolonged period of treatment that physicians tend to follow [15,16].

Although there is a plethora of data available on the matter at hand, there still exist several obstacles that limit access to fertility preservation techniques and mechanisms [14,17], while it is also notable that there are very limited data on the number of patients that are willing to adopt any of these preservation techniques. The lack of this information also affects the public health sector and the health organization system to cater to both fertility and oncology units. The authors aim to comprehensively review the published evidence on breast cancer patients and their fertility status but also identify specific treatment and nontreatment-related factors linked to the impaired infertility of these patients. 

## 2. Methodology

### 2.1. Search Strategy

This review followed the PRISMA (Preferred Reporting Items for Systematic Reviews and Meta-Analyses) guidelines. The authors conducted an extensive search of various medical databases until the 31 of July 2023. The databases utilized for the gathering of potentially relevant studies were PubMed, Embase, and the Cochrane Library.

Only the studies from the last 15 years were considered relevant due to the different modalities that were used for treating breast cancer in the past few decades and to ensure consistency in evidence across the various relevant study groups. The following terms were used for searching: “ovaria”, “ovarian”, “fertility”, “reserve”, “reservation”, “preserve”, “preservation”, “female”, “woman”, “women”, “females”, “breast”, “breasts”, “mammary”, “mammary gland”, “lymphomas”, “lymphoma”, “malignancy”, “malignancies”, “cancer”, “cancers”, “survival, “survivor”, “survivors”, ((ovaria OR ovary OR ovarian OR fertility) AND (reserve OR reservation OR preserve OR preservation)) AND ((women OR woman OR female OR females) AND ((breast OR breasts OR mammary gland OR lymphomas AND (malignancy OR malignancies OR cancer OR cancers)) AND (survival OR survivor OR survivors))). No other restrictions were applied to the query, and articles from all languages were considered.

**Figure 1:**
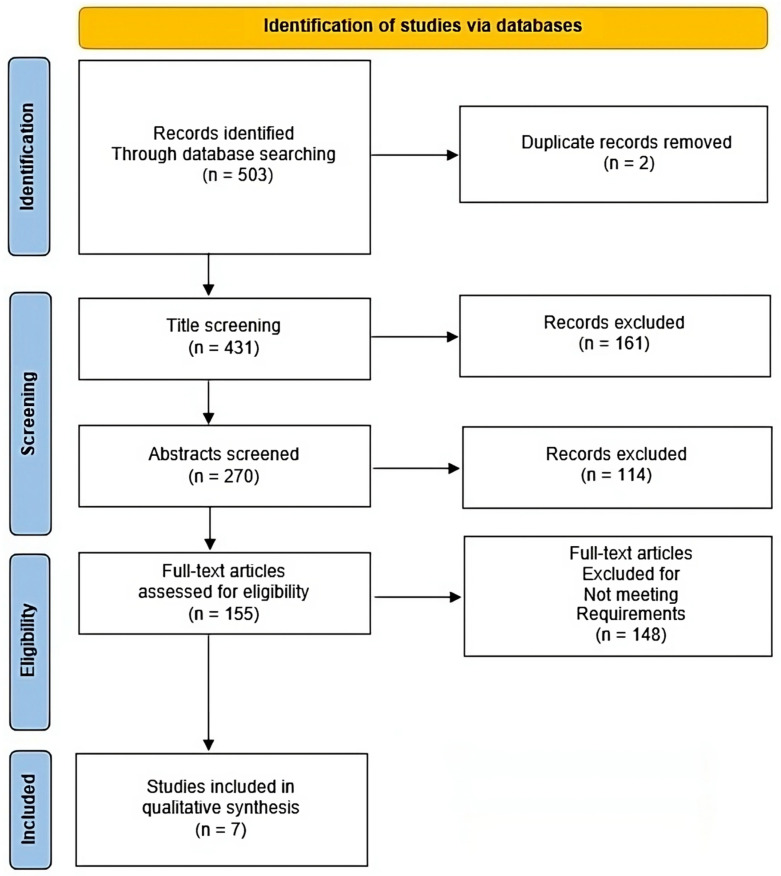
Prisma flow diagram.

The results were independently assessed by three authors (IB, MK, and KD) by reading their abstracts. If any results were considered relevant, the authors carried on reading the entire paper, and all inclusion and exclusion criteria were applied to narrow down the dataset to the relevant studies of interest. Any disagreements among authors were resolved following consensus. Furthermore, all selected papers were manually searched for relevant articles that could be of interest following the snowball procedure. The entire process can be seen in Figure 1.

### 2.2. Inclusion and Exclusion Criteria

#### 2.2.1. Inclusion Criteria 

The eligible studies that the authors considered were case–control, cross-sectional, and cohort studies, which examined the fertility or infertility status for all survivors of breast cancer through their achieving of pregnancy. Survivors were considered all patients that achieved full remission after finishing treatment and were assessed for their fertility status post-treatment.

#### 2.2.2. Exclusion Criteria 

All animal studies, cell culture studies, case reports, and case series were not considered by the authors and were excluded from the result set. Any studies that assessed the fertility status of patients straight after their breast cancer treatment was concluded were also deemed as not suitable. The authors also stumbled upon some studies with overlapping populations; in all these cases, the most up-to-date, relevant study was considered. 

### 2.3. Data Extraction and Quality Assessment 

After applying all inclusion and exclusion criteria, the result set was narrowed down to 7 relevant studies. Two authors (IB and MK) independently reviewed all articles and extracted all the data of interest using a customized data extraction form. The form included the following characteristics from all related studies: author, year of publication, type of study, period of study, country where applicable, number of patients, number of control groups where applicable, outcome variables, exposure variables, and assessment of the outcome of the study where mentioned. Based on the extracted characteristics, each study was rated either poor, fair, or good, emphasizing the sample size and the appropriate reporting of the outcome variables. 

## 3. Ovarian Suppression with Gonadotropin-Releasing Hormone Agonists

The use of Gonadotropin-releasing hormone agonists aims to lower both gonadotropins and sex hormone levels. They are commonly used to lower sex hormone levels in the treatment of hormone-sensitive cancers like breast and prostate cancers [17]. The mechanism by which ovarian suppression during chemotherapy protects ovarian function is not clear. The first studies concerned patients who received combination chemotherapy for Hodgkin’s disease and acute lymphocytic leukemia or cyclophosphamide therapy for renal diseases [18,19]. In this group, ovarian function was more disrupted in women of reproductive age than in children and young preadolescent girls.

Another proposed mechanism is the medically induced hypogonadotropic status, which reduces the number of primordial follicles that are in a differentiation state and are more susceptible to alterations by chemotherapy [20]. Accordingly, both the hypoestrogenic environment and low inhibin may prevent the increase in FSH thus protecting the follicles from atresia [21,22].

GnRH agonists have a higher potency compared to the natural GnRH molecule due to their reduced susceptibility to enzymatic degradation and higher receptor affinity. A transient release of follicle-stimulating hormone (FSH) and luteinizing hormone (LH) is caused by GnRH agonists when they bind to GnRH receptors on pituitary gonadotropin-producing cells. It usually takes 1 week of therapy for the GnRH receptors to be downregulated along with a decline in the pituitary production of both LH and FSH [23]. Several formulations of GnRH agonists are approved for parenteral administration and available on the market, including leuprolide, goserelin, triptorelin, buserelin, and histrelin.

In the last 20 years, numerous clinical studies have been conducted concerning the prophylactic administration of GnRH agonists alongside chemotherapy in women with breast cancer [24,25,26,27,28,29,30,31,32,33,34,35,36,37,38,39,40]. The authors of this article chose to refer exclusively to the studies that comment on the achievement of pregnancy for each of the fertility preservation techniques. Table 1 lists the studies that report pregnancy and examines its achievement between the group of women who underwent ovarian suppression and those who did not receive prophylactic hormone therapy.

Among the 739 patients, 66 achieved spontaneous pregnancy, of which 25 (37.87%) did not receive prophylactic agonist therapy and 41 (62.13%) did. The above results were obtained in the context of the clinical studies ZORO [27], POEMS study [35], MOFFITTs [30], OPTION [36], and PROMISE-GIM6 [29] from 2011 to 2017. In these studies, gosarelin 3.6 mg SC and triptorelin 3.75 mg IM were administered 1 to 2 weeks before the start of chemotherapy and then for up to 4 weeks during chemotherapy.

There is controversy regarding the effectiveness of GnRH agonists in achieving pregnancy. The use of gonadotropin-releasing hormone agonists (GnRHas) is still considered investigational by several authorities. Whereas previous publications have raised the fear of possible detrimental effects of GnRHa in patients with hormone receptor-positive breast cancers, recent randomized controlled trials have shown that it either improves or does not affect disease-free survival in such patients [41].

## 4. Oocyte Vitrification and Embryo Cryopreservation

### 4.1. Ovarian Stimulation Protocol

Cryopreservation of embryos or eggs is the most effective solution for preserving fertility in women with breast cancer [42]. However, oocyte retrieval is preceded by ovarian stimulation through a hyperestrogenic environment [43]. Short-term exposure to high levels of estrogens has raised concerns about the safety of conventional protocols and has led to the development of new ones that aim to counterbalance estrogen exposure in women with breast cancer undergoing ovarian stimulation for fertility preservation [44,45]. These alternative stimulation protocols consist of the addition of the selective estrogen receptor (ER) modulator tamoxifen or the aromatase inhibitor letrozole, but their effectiveness has never been compared to standard ovarian stimulation in any randomized controlled trial (RCT) [46].

Balkenende et al. in 2022 were the first to compare the effectiveness of alternatives with traditional stimulation protocols and concluded that despite the noticeable reduction in estradiol peak, alternative ovarian stimulation protocols that included tamoxifen or letrozole did not affect the number of cumulus–oocyte complexes (COCs) retrieved at follicle aspiration. There was also no evidence of a difference in the number of oocytes or embryos banked and no difference in the number of canceled cycles [47]. And the 2 antipode studies only comment that there may be a negative effect of letrozole or tamoxifen on fertilization and embryo quality in fertility preservation cycles. Further studies are needed to confirm these findings [48,49].

The most widely used protocol to stimulate patients with breast cancer is the oral administration of letrozole 5 mg or 60 mg tamoxifen from days 2–3 of the cycle. After 2 days of treatment with letrozole, a variable dose of recombinant FSH (rFSH) between 150 and 300 IU/day is added. When the concentration of serum estradiol exceeds 250 pg/mL or the follicles reach a size greater than 13 mm in diameter, administration of GnRH antagonists is started to avoid the premature peak of LH. Follicular growth is monitored until at least two of the follicles reach 20 mm in diameter, and at that moment, ovulation is triggered with the agonists of GnRH [50,51,52,53,54,55,56,57]. By comparing the use of GnRH agonists versus hCG trigger ovulation, it was found that the agonists achieved a greater and faster decline in the estradiol levels without reducing the number of mature oocytes collected or the fertilization rate [52,54,57]. This protocol with letrozole, along with final rFSH and the induction of ovulation with GnRH agonists (triptorelin), has been implemented in an extended form, independent of the molecular phenotype of breast cancer [57].

Apart from the addition of letrozole or tamoxifen to conventional stimulation protocols, alternative approaches have now been patented. A practical issue that arises in the management of these women is that in many cases, the urgency of fertility preservation does not allow the time to initiate induction early in the follicular phase. Developments in the physiology of human reproduction and the investigation of the multiple wave theory contributed in this direction, which states that the recruitment of a group of follicles is performed multiple times throughout a single menstrual cycle, enabling the initiation of ovarian stimulation at any time during the menstrual cycle [58]. The present study discusses the novel ovarian stimulation regimen, known as the random-start and luteal-phase protocol, which is distinct from the conventional stimulation approach. The development of this method represents a significant milestone in the field of fertility preservation technology, as it allows for the preservation of fertility in cancer patients without any delay in cancer therapy. It is noteworthy that the primary objective of the random-start method is cryopreservation, and hence, the challenge of utero–ovarian synchronization and preparation for embryo transfer, which is a potential pitfall of this approach, is not a concern for cancer patients. In comparison to conventional techniques, the random-start method exhibits a tendency toward a slightly elevated total dose of gonadotropin and a prolonged stimulation period during the cycle. Nevertheless, no discernible distinction in the total quantity of retrieved oocytes and mature oocytes was observed between the two approaches [59].

Finally, a promising technique that aims at obtaining a high number of oocytes in a limited time is achieved by performing two cycles of ovarian stimulation within one menstrual cycle, each at the follicular and luteal phases. Originally introduced by Kuang et al. [60], the initial oocyte retrieval is performed following the first round of stimulation. The second round begins immediately on the following day of oocyte retrieval, followed by the second oocyte retrieval. In other trials examining the efficacy of this approach, variations of the double-stimulation regimen utilizing different types and doses of gonadotropin are effective. The number of total oocytes, mature oocytes, and blastocysts in the first and second cycles was similar. More importantly, the number of total oocytes, mature oocytes, and embryos from the double-stimulation method was greater than that from the conventional cycle with a single stimulation [60,61,62].

### 4.2. Oocyte Cryopreservation

Cryopreservation of oocytes is an option for fertility preservation in women of reproductive age, who have a sufficient ovarian reserve and are diagnosed with breast cancer, regardless of the histological type of the tumor [63]. Achieving pregnancy is related to the number of mature oocytes retrieved, which is dependent on the age of the patient and her ovarian reserve at diagnosis [64,65]. A live birth rate of >40% can be estimated in women younger than 35 years, and <30% in older patients, with a very low success after the age of 40 years [66].

When the first attempts to cryopreserve eggs were made in the 1990s, the scientific community was confronted with the special cellular characteristics of the specific cells that make their freezing particularly difficult [67]. Because of their large size and limited surface area-to-volume ratio, oocytes belong to a cell category that is extremely difficult to freeze. The large amount of water in oocytes causes intracellular ice formation, chilling injury, and osmotic injury during oocyte cryopreservation [68]. Cryopreservation has also been demonstrated to have a deleterious effect on microtubule and microfilament stability, both of which are required for correct chromosomal segregation in mammalian oocytes [69]. Oocyte cryopreservation can be performed using either slow freezing (also known as equilibrium freezing) or vitrification. In the slow-freezing method, oocytes are frozen to approximately −140 °C, followed by storage in liquid nitrogen at −196 °C. These procedures usually take several hours. No serious oocyte deformation has been observed with this method, with the exception of the potential risk for ice crystal formation in the cells that may potentially affect their viability [70]. Vitrification is characterized by the instant solidification of the solution as a result of increased viscosity during cooling with higher concentrations of cryoprotectants than the concentrations used in slow freezing [71]. Nevertheless, a recent meta-analysis concluded that vitrification is superior to slow freezing for cryopreservation of both human oocytes and embryos in clinical ART [72].

Specifically for breast cancer, although there is no apparent negative influence of breast cancer diagnosis on the success of the procedure, some evidence suggests a potentially reduced performance of oocyte cryopreservation in breast cancer patients carrying germline BRCA pathogenic variants [66]. However, oocyte cryopreservation remains the first option to be discussed also in BRCA-mutated breast cancer patients [63,65]. Importantly, this strategy allows access to preimplantation genetic testing that can be of importance for these women [73].

There is a plethora of evidence in the literature regarding the technique of oocyte cryopreservation as a way of preserving fertility in young cancer patients [74]. However, the data we have from the literature only targeted at breast cancer patients are extremely limited, and the authors of this article chose to refer exclusively to them. More specifically, from the literature, we have nine clinical studies that investigate this particular technique in women of reproductive age [74,75,76,77,78,79,80,81,82], and five of them report the achievement or not of pregnancy [74,75,76,77,78]. More specifically, of the 72 women who underwent embryo transfer, 38 pregnancies were achieved (52.7%). All studies report no disease recurrence after embryo transfer. A comparison of the results between embryo cryopreservation and oocyte cryopreservation was not performed due to a lack of data from studies comparing breast cancer. Finally, it is worth noting that Alvarez et al. [76] compared pregnancy rates in women with different cancers (gynecological cancer, breast cancer, and hematological malignancies), and the case of breast cancer recorded the highest pregnancy rates.

### 4.3. Embryo Cryopreservation

Embryo cryopreservation is the most effective option for preserving fertility in women with breast cancer [74]. Oocyte retrieval and oocyte cryopreservation employ similar techniques. In embryo cryopreservation, two common methods are slow-freezing and vitrification, also utilized in oocyte cryopreservation. Cryoprotectants gradually replace cellular water through osmosis in both techniques. Slow freezing involves controlled cooling at around 2 degrees Celsius per minute, while vitrification rapidly freezes embryos in high cryoprotectant concentrations. Thawing includes removing embryos from liquid nitrogen, gradually reducing cryoprotectant concentrations, and a subsequent culture period before transfer. These methods have significantly advanced fertility preservation and assisted reproductive technologies, although staying updated with current research remains crucial in this rapidly evolving field [75].

Special attention in the case of women who survived breast cancer should be given to the embryo transfer of preserved embryos. Certain oncologists recommend that women who have successfully undergone breast cancer treatment should wait for a minimum of 2 years after diagnosis before trying to conceive. This period is crucial for monitoring any potential early recurrences [76]. However, the risk of recurrence depends on various factors, such as age at diagnosis, lymph node involvement, tumor stage, tumor biology, and hormone receptor status [77]. In addition, we must not forget that there is also the published study of the POSITIVE TRIAL, which encourages women with early hormone receptor-positive (HR+) breast cancer to stop hormone therapy after 2 years and start childbearing [78]. Hence, the decision on when to pursue conception should be personalized, considering these factors while counseling young patients about the appropriate interval between diagnosis and pregnancy. For patients with hormone receptor-positive disease, it is vital to note that tamoxifen carries teratogenic effects [79] and must not be used during pregnancy [80]. Thus, it is advisable to wait at least 2 months after completing the treatment before attempting conception [77].

There are not many studies looking exclusively at frozen embryo transfer (FET). Oktay et al. [74] in 2015 published first data regarding FET in breast cancer survivors and reported 22 pregnancies in 33 women who underwent artificial reproductive techniques. It is known from the literature that pregnancy rates after oocyte cryopreservation are similar to those after embryo cryopreservation [77]; however, studies exclusively designed in this direction only for patients who survived breast cancer are needed to draw safe conclusions.

## 5. Ovarian Tissue Cryopreservation (OTC)

OTC is a method of preserving ovarian tissue with a cryopreservation technique without the ovarian stimulation process. This method is suitable for prepubertal girls or premenarchal adolescents diagnosed with malignancy and patients unable to undergo COS because of an urgent need for cancer therapy. It is also recommended for single women who do not wish to seek a sperm donor or to freeze embryos. OTC is not considered an experimental method anymore, and it has been recognized since 2013 by the American Society for Reproductive Medicine and the Society for Assisted Reproductive Technology as one of the clinically established methods for fertility preservation [83].

The utilization of OTC offers numerous benefits, including the ability to preserve fertility in emergency situations without the need for prior ovarian stimulation protocols, while also ensuring the maintenance of both fertility and hormonal production [84]. Upon the decision to proceed with transplantation, the thawed ovarian tissue can be reimplanted either in its original location within the pelvic cavity (orthotopic transplantation) or in an alternative site (heterotopic transplantation), such as the abdominal wall or forearm. Recent studies have reported a pregnancy success rate of 26% following the transplantation of cryopreserved ovarian tissue, which encompasses both natural and IVF conceptions [85].

Again, regarding the case of breast cancer, the data we have from the literature are limited [86]. Twelve papers were found [84,85,87,88,89,90,91,92,93,94,95,96], which report a total of 24 pregnancies. Of these, three concerned case reports [88,96,97], with one of the cases concerning ovarian tissue cryopreservation in breast cancer diagnosed during pregnancy [88]. The rest of the data are derived from case series that generally concern the preservation of fertility with OCT in various malignancies. The cases were screened so that the data would have consistency exclusively for breast cancer.

## 6. In Vitro Maturation (IVM)

It is known that breast cancer cell proliferation can be induced by estrogen [98]; therefore, it is recommended to avoid high concentrations of oestradiol in these patients [99]. During IVM cycles, oestradiol concentrations are within the natural follicular phase range of up to 150 pmol/L [100], far from the super-high concentrations of oestradiol during ovarian stimulation. In this aspect, the advantage of IVM treatment for breast cancer patients is the reduced risk of stimulating estrogen-sensitive tumors, which can enhance malignant cell proliferation [101].

Initially, IVM was proposed as a means to circumvent the need for ovarian stimulation, followed by oocyte and embryo freezing. However, there is a dearth of data on the application of this method. The first clinical study on breast cancer patients was conducted in 2010, which reported pregnancy rates of 3.8% and 8.1% for oocyte freezing and embryo freezing, respectively [102]. A more recent study involving nine breast cancer patients revealed that out of 22 oocytes subjected to IVM, 12 matured successfully [103]. Furthermore, this study compared the number of mature oocytes obtained through IVM in groups of patients with different types of cancer and fertility problems. The results indicated that patients with breast cancer had a lower proportion of oocytes that matured in vitro compared to patients with other cancers and those with fertility problems (54.5% vs. 81.2% and 80.0%) [103,104]. In 2020, Malacarne et al. [105] arrived at the conclusion that there was no significant difference in the mean number of oocytes retrieved for each breast cancer patient following ovarian stimulation, as compared to healthy control women, including oocyte donors, women undergoing fertility preservation for nonmedical reasons, and female partners of infertile men participating in an in vitro fertilization (IVF) program.

Consequently, the notion of integrating IVM after ovarian tissue oocyte retrieval has garnered significant interest in enhancing fertility preservation outcomes, owing to the successful results of IVM [106]. This approach entails the maturation of immature oocytes obtained during ovarian tissue cryopreservation (OTC) through IVM, followed by their cryopreservation alongside ovarian tissue [107,108,109]. Despite the growing body of evidence on OTO–IVM, its implementation remains in its nascent stages, and its efficacy is yet to be fully established [106].

## 7. Future Perspectives

In addition to the methods mentioned above, advances in contemporary technology in the field of 3D printing and biological additive manufacturing, create expectations that in the future, we will be able to offer even more fertility preservation options to breast cancer survivors. In 2017, Laronda et al. announced the creation of the first 3D-printed functional ovarian implant in a hydrogel scaffold [110]. Following this specific scientific achievement, Wu et al. in 2022 expanded the research of biological materials that can be used as bioinks in bio 3D printers and, finally, printed a gelatin–methacryloyl scaffold for ovarian implants [111]. The data are currently limited and all experimental; however, the technology is making significant progress, and clinical practice must follow and incorporate it, for the benefit of the patients.

## 8. Discussion

Infertility among young female breast cancer patients still has a big impact and a negative outcome as a result of treatment of the disease. Most of these women will be advised to undergo adjuvant chemotherapy with the assistance of antihormonal therapy in some instances to minimize the risk of death or recurrence of the tumor. The gonadotoxicity of adjuvant chemotherapy, especially if it is performed with alkylating agents, like cyclophosphamide, and the fact that it can accelerate the rate of primordial follicle loss or decrease the reserve of primordial follicles for a patient can be catastrophic for them and cause infertility and thus any physiological or psychological problems related to it.

Multiple factors can affect ovarian failure following breast cancer treatment, namely the age of a patient, the type and dosage of chemotherapy, the number of cycles of chemotherapy, their ovarian reserve before treatment, etc. These patients must have an assessment of their fertility status before undergoing therapy. In developed countries, this is already standard practice with hormonal evaluation tests or even descriptive statistics. Educating the patients accordingly and keeping family history data along with annual mammography screening and chemopreventative drugs have led to higher survival rates, fewer deaths, and lower occurrences. Maintaining fertility after breast cancer treatment for these patients is very important. Scientists are looking into new ways of preserving a patient’s fertility by ovarian stimulation with different agents, like letrozole, FSH, and tamoxifen, in order to minimize the impact that elevated serum estrogen levels can have on the growth of the tumor.

Oncofertility counseling is now a concept that is catching on and allows patients to be informed on the options they have in order to be able to live a normal life and have their own offsprings after they are finished with the cancer treatment. Being educated and appropriately counseled is required. A multidisciplinary approach should be taken and appropriate planning of the treatment should occur, with free access to resources and information in order to have a better chance against infertility. Establishing rapid fertility consultation links with programs related to the survival of breast cancer could help in ensuring that all these young women who would possibly lose their fertility due to the gonadotoxic chemotherapy treatment are counseled for the adherent effects of it and have an increased likelihood of childbearing after cancer treatment.

On the bright side though, these new techniques that scientists are looking into have very positive outcomes, with tests indicating that offspring born by breast cancer survivors have no congenital abnormalities and can live a healthy life. Although currently we are passing through a period of change and uncertainty regarding the variety of fertility preservation mechanisms for young breast cancer survivors, the evolution and development of new techniques in the near future promise to be very exciting.

## Figures and Tables

**Table 1:** Studies that report pregnancy and examine the achievement between two groups. Group A: women who underwent ovarian suppression; Group B: women who did not receive prophylactic hormone therapy.

Authors	Publication Year	Number of Patients	Pregnancies (GnRH vs. Control)
Gerber et al. [27]	2011	60	1 vs. 1
Del Mastro et al. [29] and Lambertini et al. [15]	2011, 2016	281	8 vs. 3
Munster et al. [30]	2012	49	0 vs. 2
Elgindy et al. [31]	2013	100	2 vs. 1
Moore et al. [35]	2015	218	23 vs. 13
Leonard et al. [36]	2017	221	7 vs. 5

## Data Availability

Not applicable.
